# Dr. Gardner Quincy Colton

**Published:** 1898-10

**Authors:** C. S. McNeille


### Obituary.

DR. GARDNER QUINCY COLTON, through whose instumentality "Nitrous Oxide Gas" was first used in dentistry, died Aug. 9th, 1898, in Rotterdam, Holland. He had been on a visit to Europe and was about to return home when he succumbed to a complication of diseases brought on by old age.

Dr. Colton was born in Georgia, Vt., Feb. 7th, 1814, and was the twelfth child of his parents. He first learned chair making and when twenty-one years old came to New York where he followed his trade, studying all the time, however, in the hope of becoming a physician. In 1842 he entered the College of Physicians and Surgeons, and later studied in the office of the late Dr. Williard Parker.

Two years after he began to deliver lectures on physiology and chemical phenomena. He had acquired a knowledge of electricity, a science then in its infancy, and invented an electric motor which he exhibited, illustrating his lectures with it. This motor is now in the Smithsonian Institute in Washington.

Dr. Colton went to California in 1849, where he searched for gold and practiced medicine among the miners. He was the first man in California to be appointed a Justice of the Peace. With a competence, he returned to the East and went about the country lecturing telling his audiences of the anæsthetic properties of the laughing gas. In 1863 he established an office in the Cooper Institute. A few years later was able to visit Paris with a record of 20,000 administrations. Returning to America he opened offices in Philadelphia, Boston, Baltimore, and several other cities, and thus, through his energy and success the use of Nitrous Oxide gas as an anæsthetic became thoroughly established and dentists throughout the length and breadth of the land began to use it, and it dates from the re-discovery in 1863.

Away back in 1844, when he was lecturing in Hartford, Conn., and showing the effects of Nitrous Oxide gas on persons to whom he administered it on the stage, Dr. Horace Wells, who became one of his subjects, was impressed with the possibility of using the gas in dentistry. He told Dr. Colton of his idea, and the next day he had the gas administered to him and a tooth extracted.

Dr. Colton was also an author and a Shakespearean scholar. He published a brochure on Shakespeare and the Bible, and wrote a good deal upon the discovery of anæsthesia.

C. S. McNEILLE, D. D. S.

---

